# Enhanced COVID-19 Detection from X-ray Images with Convolutional Neural Network and Transfer Learning

**DOI:** 10.3390/jimaging10100250

**Published:** 2024-10-13

**Authors:** Qanita Bani Baker, Mahmoud Hammad, Mohammed Al-Smadi, Heba Al-Jarrah, Rahaf Al-Hamouri, Sa’ad A. Al-Zboon

**Affiliations:** 1 Faculty of Computer and Information Technology, Jordan University of Science and Technology, P.O. Box 3030, Irbid 22110, Jordan; m-hammad@just.edu.jo (M.H.); haaljarah18@cit.just.edu.jo (H.A.-J.); rmalhammouri18@cit.just.edu.jo (R.A.-H.); saalzboon16@cit.just.edu.jo (S.A.A.-Z.); 2 Digital Learning and Online Education Office (DLOE), Qatar University, Doha 2713, Qatar; malsmadi@qu.edu.qa

**Keywords:** deep learning, convolutional neural networks, CNNs, transfer learning, X-ray, medical images, COVID-19

## Abstract

The global spread of Coronavirus (COVID-19) has prompted imperative research into scalable and effective detection methods to curb its outbreak. The early diagnosis of COVID-19 patients has emerged as a pivotal strategy in mitigating the spread of the disease. Automated COVID-19 detection using Chest X-ray (CXR) imaging has significant potential for facilitating large-scale screening and epidemic control efforts. This paper introduces a novel approach that employs state-of-the-art Convolutional Neural Network models (CNNs) for accurate COVID-19 detection. The employed datasets each comprised 15,000 X-ray images. We addressed both binary (Normal vs. Abnormal) and multi-class (Normal, COVID-19, Pneumonia) classification tasks. Comprehensive evaluations were performed by utilizing six distinct CNN-based models (Xception, Inception-V3, ResNet50, VGG19, DenseNet201, and InceptionResNet-V2) for both tasks. As a result, the Xception model demonstrated exceptional performance, achieving 98.13% accuracy, 98.14% precision, 97.65% recall, and a 97.89% F1-score in binary classification, while in multi-classification it yielded 87.73% accuracy, 90.20% precision, 87.73% recall, and an 87.49% F1-score. Moreover, the other utilized models, such as ResNet50, demonstrated competitive performance compared with many recent works.

## 1. Introduction

The COVID-19 pandemic has emerged as a critical global health challenge that has caused an urgent global health crisis. Designated by the World Health Organization (WHO) [1], this virus, formally known as Coronavirus, represents a highly contagious and perilous respiratory infection. Originating in Wuhan, China in November 2019, COVID-19 rapidly spread across the globe, earning its status as a pandemic. Its rapid transmission elevated it to the forefront of global medical concerns.

Early diagnosis of COVID-19 plays a critical factor in controlling its spread and mitigating its impact on public health, especially in regions with a rapid increase in confirmed cases. In this critical context, medical imaging techniques, particularly the analysis of Chest X-ray images, have demonstrated potential as an invaluable tool for the disease diagnostic process [2,3]. Recently, Deep Learning (DL) techniques [4] have led to remarkable advances in medical data analysis, including medical images, wherein DL techniques provide huge potential for extracting tiny features in image analysis [5], particularly in the context of COVID-19 [4].

For this study, we collected data from two different public resources: the first was the Extensive and Augmented COVID-19 X-ray and CT Chest Images Dataset [6], and the second was the Kaggle site, which was originally collected from [7]. The collected datasets each consisted of 15,000 X-ray images. In this research, we divided the work into two main tasks: the first task was the binary classification task, which was related to classifying the given X-ray images into Normal or Abnormal, while the second task was a multi-class classification task, where the goal was to classify the X-ray images into one of three classes: Normal, COVID-19, or Pneumonia.

This work included several new non-trivial extensions to the preliminary version from our research in [8]:

1. In this work, we used larger datasets. We increased the number of images in this work to 15,000 X-ray images, while in our previous work [8] we had a smaller dataset, which was 7800 images. Also, in this work we had balanced datasets, which meant that the number of samples in each class was equal, while in our previous work the dataset was imbalanced. In [8], the employed dataset was smaller and imbalanced, leading to inflated performance metrics. By using balanced and larger datasets in this study, the models faced a more challenging classification task, which resulted in more realistic and reliable performance metrics.
2. In this research, we used data pre-processing techniques that differed from the previously used techniques in [8]. We deployed more data-augmentation techniques, such as image color conversion (from gray-scale image to RGB image) using the OpenCV [9] Python 3.10 Google Colab version. library. In addition, in this research, we used different types of data augmentation to increase the number of images that were used to train the proposed models to detect COVID-19 more accurately. The total number of images after data augmentation was 553,500 images.
3. We also utilized deep learning architectures that differed from the previously used architectures. In the previous work, we limited the used approaches to Inception-V3, Xception, and MobileNet, focusing only on the binary task, while in this work we utilized six distinct CNN models (Xception, Inception-V3, ResNet50, VGG19, DenseNet201, and InceptionResNet-V2) for both binary (Normal vs. Abnormal) and multi-class (Normal, COVID-19, Pneumonia) classification tasks.
4. The utilized models are promising, as they achieved highly competitive results, compared to the previous research, of 98.13% accuracy, 98.14% precision, 97.65% recall, and a 97.89% F1-score in binary classification, while in multi-classification they yielded 87.73% accuracy, 90.20% precision, 87.73% recall, and an 87.49% F1-score. Compared with the recently published work, the obtained results outperformed the others clearly in multiclass classification, especially for the ResNet50 and Xception models.
5. In addition to the foregoing technical contributions, we have conducted a literature review, and we discuss many related research efforts in this area.

The remainder of this paper is structured as follows. Section 2 covers the related work. Section 3 describes the applied methodology, the dataset, and how we utilized several transfer models for the two classification tasks. Section 4 describes the different evaluation metrics we used to compare the various machine learning classifiers and covers the evaluation design and results. Section 5 provides a detailed discussion on the model performance compared with existing works, the role of data augmentation on the model performance, the potential in the real-world applications, and clinical integration, as well as, in the section, the limitations and future directions of the proposed approach are also discussed. Section 6 discusses the research findings and concludes the paper with avenues for future work.

## 2. Literature Review

Recently, the power of deep learning technologies has been applied in the effective and timely detection of COVID-19 from different image types [10]. In this section, we delve into a comprehensive overview of previous studies focused on utilizing X-rays to detect COVID-19. Several surveys have provided a comprehensive overview of the approaches used in COVID-19 detection using different types of medical images. In [4], Bhosale et al. presented a systematic review of the recent DL techniques utilized to classify COVID-19 from different lung and chest imaging. Other systematic reviews have recently been conducted in [11,12,13].

Some previous studies have leveraged CT scan images for the detection of COVID-19. Zheng et al. proposed DeCoVNet [14] to detect if the patient is infected with Coronavirus or not, depending on CT images. Firstly, they used a UNET pre-trained model for segmented lung regions then used a 3D deep neural network for prediction. To evaluate the proposed software, they collected CT images for the train and test sets from 13 December 2019 to 6 February 2020 and used data augmentation to increase the dataset size, and the ROC AUC achieved was 0.959. In [15], Li et al. proposed a method to distinguish between COVID-19, Community-Acquired Pneumonia (CAP), and other non-pneumonia, using transfer learning techniques dependent on chest CTs. In the proposed method, they used UNET for segmentation, which used ResNet50 to extract features, then they fed these features into max pooling and, finally, they fed them into a fully connected layer and softmax activation function for prediction. The dataset used to evaluate this method was collected in six hospitals from 3322 patients, and it contained 4356 chest CT images. They found that the deep learning model demonstrated high accuracy in detecting COVID-19 and effectively distinguishing it from Community-Acquired Pneumonia and other lung diseases, where the AUC of COVID-19 was 0.96. Shi et al. [16] aimed to find a deep learning model that would predict in cases of people with Coronavirus their symptoms and severity, based on the CT and initial clinical features. They used MOICT with some features that they obtained from health organizations, and they used the LASSO logistic regression model, which achieved good results, as the accuracy reached 0.890, which was higher than other manual results, like using MOICT, POICT, or PSI.

Moreover, Chen et al. [17] utilized a deep learning model to detect pneumonia in COVID-19 patients, using CT scan images. In addition, they aimed to reduce the workload of radiologists. Their dataset was collected from Renmin Hospital, Wuhan University, where they collected 46,096 CT images from 106 patients, including 51 patients infected with COVID-19 pneumonia and 55 control patients of other diseases. They used the UNet++ model applied to 289 randomly selected CT images. These images had been labeled by experts, to find the intact area of the image in CT images, and the process of testing the UNet++ was conducted on other other randomly selected CT images. The UNet++ model achieved excellent results on the testing data, where the accuracy reached 100%. Song et al. [18] proposed a deep learning model to detect COVID-19-infected patients from CT (Computed Tomography) images. They collected the CT images from more than one hospital; the dataset used contained 88 CT scan images of COVID-19 patients, 101 infected with other viruses’ pneumonia, and 86 healthy persons. In their approach, they used the DRE-Net model and achieved a high result on AUC of 0.99 and accuracy of 0.94 on the test set. Wang et al. [19] proposed to find a deep learning model that could check up and diagnose the COVID-19 pneumonia patient based on CT scan images. Their dataset consisted of 453 CT images from infected patients and healthy persons. They used the Inception model as a baseline and added some enhancements, to enable the model to be learnable in the last layers. The proposed model achieved accuracy of 82.9%. Xu et al. [20] proposed a deep learning model that would examine the COVID-19 pneumonia patient based on CT scan images. Their dataset consisted of 618 CT images of patients with COVID-19, Influenza-A viral pneumonia, and healthy persons. They used the location-attention-oriented model and achieved accuracy of 86.7%.

On the other hand, numerous studies have used X-ray images to detect COVID-19, employing various approaches. Prabira et al. [21] proposed a transfer learning technique that consisted of Resnet50 and SVM, to classify COVID-19 using X-ray images. The ResNet50 model was selected after testing eight pre-trained models, including AlexNet, VGG16, VGG19, Google Net, ResNet50, ResNet101, and XceptionNetin order, to use the best. The authors used Resnet50 to extract deep features and then used SVM for classification. Finally, the proposed method was evaluated, using datasets collected from GitHub, Kaggle, and OpenAI. The accuracy achieved was 95.38%. To detect pneumonia using X-ray images, Rajpurkar et al. [5] proposed an algorithm called CheXNet. They trained the dataset on a 121-layer Convolutional Neural Network. The CheXNet dataset, trained on the ChestX-ray14 dataset, contains about 112,120 frontal-view X-ray images with 14 different classes, including pneumonia. Gozes et al. [22] proposed an AI-based tool that uses CT images for Coronavirus detection. This tool combines 2D and 3D deep learning models. They applied segmentation techniques to extract lung regions and then trained six different datasets for patients from China and the U.S. on ResNet50-2D. The sensitivity and specificity achieved were 98.2% and 92%, respectively.

Nahiduzzaman et al. [23] proposed a novel model called the Chest X-ray6 model, which depends on the lightweight CNN model to detect five diseases, including COVID-19. They applied their approach to 9514 Chest X-ray images, which they collected from different databases with six classes. The dataset was unbalanced, they applied five different augmentation techniques, and the number became 21,000 images. Finally, they had two classification tasks: binary and multi-classification. Their model achieved accuracy of 97.94% and 80% for both tasks, respectively. In [24], Constantinou et al. aimed to study the possibilities of a deep learning-based approach to detecting the COVID-19 disease. They focused on five models: ResNet50, ResNet101, DenseNet121, DenseNet 169, and InceptionV3. They evaluated their developed models on the COVID-QU dataset, which contains 33,920 X-ray images in three classes: COVID-19, Non-COVID-19, and Normal. The result showed that ResNet101 outperformed the other models, with accuracy of 96%.

In [25], Dawar et al. developed a system that could distinguish the COVID-19 X-ray disease from others. The dataset used was collected from an open repository that contained 15,000 images categorized into COVID-19, Pneumonia, and Normal. Four Convolutional Neural Network models were used: VGGNet, LeNet5, AlexNet, and their custom model, which consisted of five convolutional layers followed by four dense layers. The result showed that the custom model performed best compared to the other models, with accuracy reaching 93.96%. In [26], Chakraborty et al. proposed a deep learning model dependent on the ResNet18 pre-trained model. First, they collected 10,040 X-ray images from different open sources, like Kaggle and GitHub, to detect COVID-19 images among images classified as pneumonia and normal. Then, they applied their preprocessing steps and fed them into the model. Their model achieved accuracy of 96.43% and sensitivity of 93.68%.

Gupta et al. [27] developed a system dependant on X-ray images to detect COVID-19 diseases. They used an open-source dataset with 2905 images labeled into three classes: COVID-19, viral pneumonia, and healthy. The deep learning models used in this study were VGG16, MobileNetV2, ResNet18, and AlexNet. They concluded that the AlexNet model outperformed the other models, with accuracy of 97.6%. In [28], Dhiman et al. developed 11 Convolutional Neural Network (CNN)-based models—AlexNet, VGG16, VGG19, GoogleNet, ResNet18, ResNet50, ResNet101, InceptionV3, InceptionResNetV2, DenseNet201, and XceptionNet—to detect COVID-19 using X-ray images by classifying them into COVID-19 and normal. The dataset used was collected from open repositories like GitHub and Kaggle. They found that ResNet101 with the J48 decision tree classifier outperformed the other models, with accuracy of 98.54%. In [29], Narin et al. proposed five transfer learning methods based on pre-trained models (ResNet50, ResNet101, ResNet152, InceptionV3, and Inception-ResNetV2). They applied their models on three different binary datasets: 1-(COVID-19 and Normal), 2-(COVID-19 and Viral Pneumonia), and 3-(COVID-19 and Bacterial Pneumonia), using X-ray images. They concluded that the ResNet50 model achieved the best results among the models on the three datasets, with accuracy for dataset 1: 96.1%, dataset 2: 99.5%, and dataset 3: 99.7%, respectively. In [30], Ozturk et al. proposed a deep learning model based on the Darknet-19 model, with some modifications to the number of filters and convolutional layers, named the DarkCovidNet model. Their study was based on X-ray images collected from two different resources with three classes. They conducted their study on two classification tasks: binary (COVID and no-findings) and multi-classification (COVID, no-findings, and pneumonia). Their model achieved accuracy of 98.08% and 87.02% for both tasks, respectively. Enas in [31] utilized a deep learning framework for early COVID-19 diagnosis using Chest X-ray images, with preprocessing for image enhancement and a classification phase applying pre-trained Convolutional Neural Network models (VGG19 and EfficientNetB0). The best model achieved high sensitivity of 0.96, specificity of 0.94, precision of 0.9412, an F1 score of 0.9505, and accuracy of 0.95 for binary classification of COVID-19 and normal Chest X-rays, and classification accuracy of 0.935 for a four-class classification. Recently, [32] explored the effectiveness of several deep learning models, including Xception, VGG-16, and ResNet. Their work utilized two datasets: the first comprised 4050 X-ray images, and the second had 6378 images. Their results demonstrated that the Xception-Enhanced Model achieved precision of 98.8%, significantly outperforming the ResNet50 model, which had precision of 60%. The standard Xception model and VGG-16 also performed well, with precisions of 86.74% and 92%, respectively.

Table 1 provides a summary of the prior research discussed in this study.

Recently, several studies in the detection of COVID-19 from Chest X-rays have been introduced, utilizing different deep learning models. Bukhari et al. [33] utilized DenseNet169, demonstrating validation accuracy of 100%, outperforming the ResNet and VGG models. Roy et al. [34] proposed a model combining Xception, InceptionV3, and ResNext50, resulting in accuracy of 98.44%, which showed a 4.44% improvement over prior studies. Comparing the proposed model in this work, the utilization of Xception for both binary and multi-class classification achieved similar binary classification accuracy of 98.13% and multi-class accuracy of 87.73%. The results obtained in our research illustrate the proposed approach’s competitiveness with other state-of-the-art models, especially for multi-class classification tasks, where our model performed comparably to more complex architectures. Moreover, Ramkumar et al. [35] have proposed a new approach that combines MobileNetv1 with Jellyfish Search Optimization for COVID-19 detection. The proposed method includes multi-head attention mechanisms that improve precision and computational efficiency. However, our proposed model maintains high accuracy and recall without requiring additional optimization techniques, which reinforces the model’s simplicity and robustness. Henna et al. [36] also used transfer learning techniques, with CLAHE-based data augmentation, to train models like AlexNet and VGG16 on smaller datasets. Our proposed Xception-based model with extensive data augmentation achieved superior results on a much larger dataset, which shows the scalability and efficacy of our proposed approach. More recent proposed models, such as Singh et al. [37], used VGG16 with transfer learning for COVID-19 detection, showing strong results in feature extraction using data augmentation and pre-trained weights. Ali et al. [38] utilized a modified CNN with k-Nearest Neighbor to classify COVID-19 severity, reporting 92.80% testing accuracy. Meanwhile, Rashed et al. [39] utilized a Conditional Cascaded Network (CCN) with transfer learning, showing high precision and specificity using multiple datasets, while Khattab et al. [40] integrated focal loss with several deep learning models, like InceptionResNet V2 and Xception, and they showed classification accuracy of up to 100% on some datasets. While these proposed works emphasized the adaptability of transfer learning and optimization techniques, our proposed model combines Xception and data augmentation, yielding 87.73% multi-class accuracy and 90.20% precision, thus remaining highly competitive in both performance and simplicity. Additionally, the work proposed by Rashed et al. [39] employed a CNN approach for COVID-19 diagnosis using Chest X-rays and CT images, demonstrating robust performance metrics across different architectures. Moreover, the work of Khattab et al. [40] combined transfer learning models and data-mining techniques for class imbalance. These comparative results indicate that while several state-of-the-art techniques utilize complex multi-model architectures or novel optimization methods our proposed approach remains competitive, with a focus on simplicity, transfer learning, and effective data augmentation.

**Table 1:** Overview of related research in COVID-19 detection using medical imaging.

Ref.	Year	Image Type	Approach	Dataset	Results
[40]	2024	X-ray	Four models: InceptionResNet V2, MobileNet Inception V3, and Xception.	Four public datasets.	For the first dataset, the InceptionResNet V2 was 88.63%. For the second and fourth datasets, the Inception accuracy was 94.35% and 97.67%.
[32]	2024	X-ray	Xception, VGG-16, and ResNet.	Two datasets: the first one comprises 4050 images; the second one has 6378 images.	Precision for Xception-Enhanced Model (98.8%), ResNet50 (60%), Xception (86.74%), and VGG-16 (92%).
[31]	2024	X-ray	CNN models.	10,192 Normal cases, 3616 positive COVID-19 cases, 1345 Viral Pneumonia cases, and 6012.	Sensitivity of 0.96, specificity of 0.94, precision of 0.9412, F1 score of 0.9505 and accuracy of 0.95.
[23]	2023	X-ray	Lightweight CNN model.	9514 Chest X-ray images from different databases.	Accuracy for binary classification = 97.94% and for multi-classification task = 80%.
[24]	2023	X-ray	ResNet101.	33,920 X-ray images called COVID-QU-Ex.	Accuracy = 96%.
[25]	2023	X-ray	Customized model consisting of five convolutional layers followed by four dense layers.	15,000 X-ray images from open-source repositories.	Accuracy = 93.96%.
[26]	2022	X-ray	ResNet model.	10,040 X-ray images from different open-source repositories.	Accuracy = 96.43%, Sensitivity = 93.68%.
[21]	2020	X-ray	Transfer learning technique consists of Resnet50 and SVM.	381 X-ray images from different open-source repositories.	Accuracy = 95.38%.
[5]	2017	X-ray	CheXNet algorithm.	ChestX-ray14 dataset.	CheXNet outperforms radiologists and previous state-of-the-art models.
[22]	2020	CT	Segmentation techniques to extract lung region, and ResNet50-2D for classification.	6150 CT slices of 157 international patients (China and U.S.).	Sensitivity = 98.2% and specificity = 92.2%.
[14]	2020	CT	Proposed software system called (DeCoVNet), using UNET for segmentation and 3D deep neural network for classification.	Collected CT images.	ROC AUC = 0.959
[15]	2020	CT	Transfer learning techniques (UNET, ResNet50, max pooling, fully connected layer, and softmax activation function).	Collected in six hospitals from 3322 patients; contained 4356 chest CTs.	AUC = 0.96.
[16]	2020	CT	Based on MOICT and used the LASSO logistic regression model.	CT images of a total of 196 patients.	Accuracy = 89%
[17]	2020	CT	Detecting COVID-19 patients using CT images by using UNet++ model.	46,096 CT images.	Accuracy = 100%.
[18]	2020	CT	DRE-Net model.	Dataset containing 88 CT scan images of COVID-19 patients.	Accuracy = 94%, AUC = 0.99.
[19]	2020	CT	Using the Inception model.	453 CT images from infected patients and healthy persons.	Accuracy = 82.9%.
[20]	2020	CT	Using the location-attention-oriented model.	618 CT images from COVID-19 and Influenza-A viral pneumonia patients and from healthy persons.	Accuracy = 86.7%.

Recently, Large Language Models (LLMs) [41] have taken the spotlight in the natural language processing domain. Furthermore, the integration between LLMs and vision enables the users to explore emergent abilities with multimodal data [42]. Recent advances in Vision–Language Models (VLMs) have demonstrated significant potential for medical imaging analysis and diagnosis tasks [43]. Lozano et al. introduced μ-Bench, which is a benchmark designed to evaluate the performance of VLMs in microscopy tasks, highlighting the challenges of applying these models in distinguishing between microscopy modalities and domains. Their work revealed that even state-of-the-art VLMs struggle with basic biomedical tasks, which underscores the need for further model development to enhance their utility in medical applications [44]. Moreover, in [45] Moon et al. developed MedViLL, which is a BERT-based model tailored for vision–language tasks in radiology, achieving superior performance in diagnosis classification and medical image report retrieval. MedViLL shows the potential for multimodal approaches to improving the generalizability and interpretability of AI models in the medical field. Radford et al. [46] introduced the use of vision–language models, like CLIP, that leverage large-scale datasets of image–text pairs, to enable the zero-shot learning utilized in various computer vision tasks. Their approach has inspired applications in medical imaging, as in the work developed by Huang et al. [47], where PLIP—a pathology-specific vision–language model was trained on a large dataset of images from medical Twitter. PLIP demonstrated state-of-the-art performance in pathology image classification, especially in zero-shot scenarios, illustrating the adaptability of VLMs in medical diagnosis. Zhang et al. [48] examined the performance of popular multimodal LLMs, such as GPT-4 and Gemini, for a variety of medical imaging tasks, noting strengths in report generation and lesion detection. Panagoulias et al. [49] evaluated utilizing GPT-4’s diagnostic accuracy in pathology, demonstrating promising results but highlighting specific weaknesses in the model’s knowledge graph integration and entity recognition. Generally, the integration of vision–language models in the medical imaging domain is still in its early stages, with ongoing research and efforts to improve their robustness and applicability in clinical settings and analysis tasks.

## 3. Research Methodology

In this section, we discuss the proposed framework in Section 3.1; then, we present the datasets employed to train and evaluate the proposed model in Section 3.2. In Section 3.3, we present the data-augmentation techniques [50] applied to increasing the number of X-ray images and the data diversity to enhancing the performance and generalization of the models utilized in this research. Finally, Section 3.4 provides a detailed discussion of the models utilized in this research.

### 3.1. Pneumonia Detection Framework

**Figure 1:**
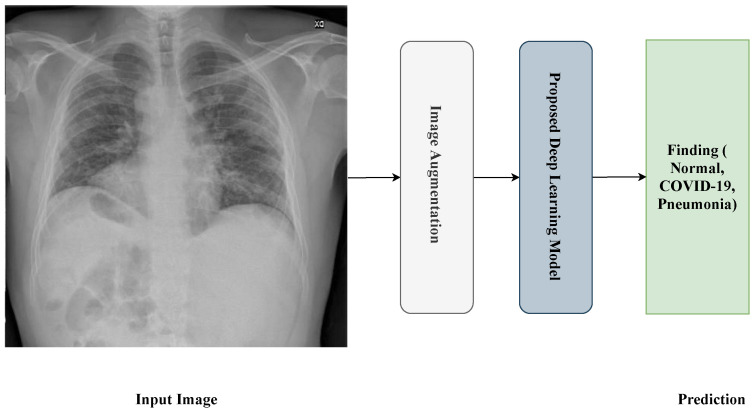
General framework of the proposed approach.

Figure 1 shows the proposed framework for the detection process. The framework passes through four steps:

1. Reading and resizing process of the X-ray image.
2. Image-augmentation process.
3. Training process.
4. Prediction process.

### 3.2. Dataset

**Figure 2:**
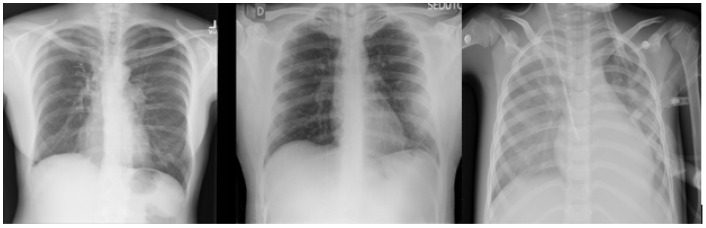
X-ray images of Normal, COVID-19, and Pneumonia used in binary classification task.

The datasets used in this research were collected from two datasets: the first was from [6], titled “Extensive COVID-19 X-ray and CT Chest Images Dataset” (Available online: https://dx.doi.org/10.17632/8h65ywd2jr.2 (accessed on 25 June 2023 )); the second was from the Kaggle site (Available online: https://www.kaggle.com/paultimothymooney/chest-xray-pneumonia (accessed on 12 June 2023)) that was obtained from [7]. In the collection step, we considered a focus on having a balanced dataset by ensuring that each class comprised almost the same number of X-ray images, each having 5000 X-ray images. Figure 2 shows an example of the collected dataset (X-ray images) with three classes: Normal, COVID-19, and Pneumonia, respectively, from left to right.

#### 3.2.1. Binary Classification Task Dataset

**Figure 3:**
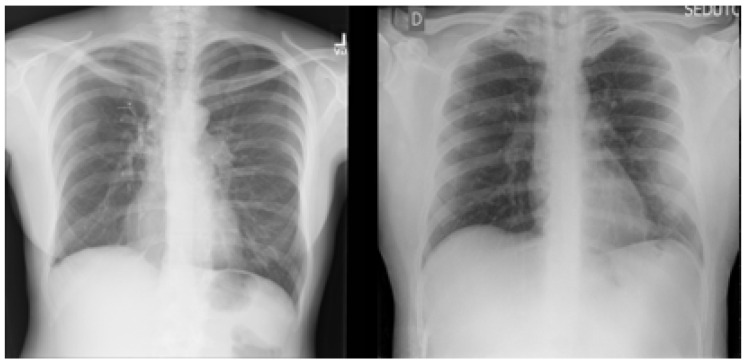
X-ray images of Normal and Abnormal.

For the binary classification task used in this research, the dataset consisted of 15,000 X-ray images, labeled with two classes: (1) Normal (which meant that the patient was healthy and did not suffer from any pneumonia type); and (2) Abnormal (which meant that the patient was suffering from pneumonia because of Coronavirus or other types of viruses). Figure 3 shows an example of the dataset (X-ray images) classes used for the binary classification task: Normal and Abnormal, respectively, from left to right.

Table 2 illustrates the number of X-ray images used to train and evaluate the proposed model.

#### 3.2.2. Multi-Class Classification Task Dataset

For the multi-class classification task used in this research, the dataset consisted of 15,000 X-ray images, labeled with three classes: (1) Normal (which meant that the patient was healthy and was not suffering from any pneumonia type); (2) COVID-19 (which meant that the patient was suffering from pneumonia because of Coronavirus); and (3) Pneumonia (which meant that the patient was suffering from pneumonia because of another type of virus, like SARS). Figure 2 shows an example of the dataset (X-ray images) classes used for multi-class classification tasks: Normal, COVID-19, and Pneumonia, respectively, from left to right.

Table 3 illustrates the number of X-ray images for each class regarding the training and testing process of the multi-class classification task.

### 3.3. Dataset Augmentation

Data augmentation is a technique used to enlarge the number of samples of the training dataset by performing operations, such as scaling, rotation, and flipping. The goal of this technique is to match the vast amount of data needed to train large and complex deep learning-based models, as we discuss in Section 3.4.

**Figure 4:**
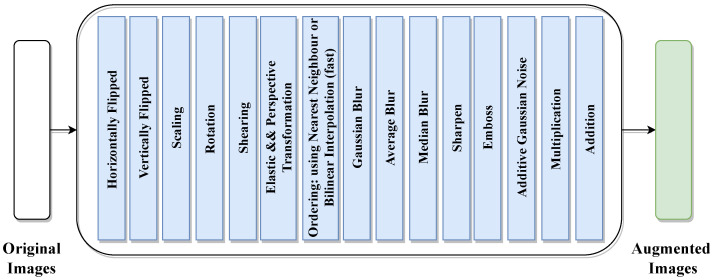
Image-augmentation techniques.

As shown in Figure 4, we applied several data-augmentation techniques, such as scaling, rotation, multiplication, addition, horizontal and vertical flipping, Gaussian blur, shearing, and so on. After applying the augmentation techniques, the number of images reached 216,000 images; we computed this number by multiplying the number of original training images by the number of augmentation techniques used, which was 15. Finally, we added the results to the number of original training images number ((13,500 × 15) + 13,500 = 216,000). Table 4 illustrates the description of the image-augmentation techniques used.

### 3.4. Proposed Approaches

In this research, we used transfer learning techniques based on different pre-trained deep learning models. In this section, we discuss the Xception, Inception-V3, ResNet, VGG, DenseNet, and InceptionResNet-V2 models. In Section 3.4.7, we discuss the layers of transfer learning technique utilized.

#### 3.4.1. Xception Model

The Xception model, as proposed in [51], is a model that was released by Google to be a new architecture in Convolutional Neural Networks. Its idea was inspired by the Inception model but with some modifications to depthwise separable convolution. The Xception model consists of 36 convolutional layers stacked in 14 modules, all of which have a linear residual connection except in the first and last modules, which enables the model to achieve high accuracy. The results proved that Xception is superior to the state-of-the-art models (e.g., Inception-V3 [52], VGGNet [53], and ResNet [54]). Table 5 illustrates the evaluation results (Top-1 and Top-5 accuracy) of the Xception model on ImageNet [55].

#### 3.4.2. Inception-v3 Model

The Inception-V3 model [52] is a very deep CNN model, which is built from 11 inception modules. Each module contains a convolutional filter and pooling layer. After the modules, the Inception-v3 model has a dropout layer and then three fully connected layers. This model feeds two-dimensional images. Table 6 illustrates the evaluation results (Top-1 and Top-5 accuracy) of the Inception-v3 model on ImageNet [55].

#### 3.4.3. ResNet50 Model

ResNet is a powerful neural network, and it is considered a backbone for computer vision tasks and other recognition tasks. The main idea of ResNet is the skip connection, to avoid the vanishing gradients problem [54]. The model is considered a starting point for a transfer learning model, due to its easy use. The ResNet model has several versions with the same idea but with a different number of layers, like ResNet-50, ResNet-101, and ResNet-152. Table 7 illustrates the evaluation results (Top-1 and Top-5 accuracy) of the ResNet model on ImageNet [55].

#### 3.4.4. VGG Model

VGG [53] is a deep Convolutional Neural Network released by a group from Oxford University used for large-scale image recognition. The main idea of the VGG model is to increase the depth of the network with the use of very small filters (3 × 3). The VGG model has two versions, VGG-19 and VGG-16: each number denotes the number of weight layers. Table 8 illustrates the evaluation results (Top-1 and Top-5 accuracy) of the ResNet model on ImageNet [55].

#### 3.4.5. DenseNet Model

DenseNet [56] is a neural network that has established a new state-of-the-art result in visual object recognition tasks. DenseNet is similar to the ResNet model, but has some differences. DenseNet uses concatenation between the output of the previous layer with the next layer, while ResNet uses an additive approach. Also, DenseNet uses fewer parameters than ResNet. The main advantages of the DenseNet model are the re-use feature, a reduced vanishing gradient problem, and fewer parameters. DenseNet model has several versions: DenseNet-169, DenseNet-121, and DenseNet-201; each number denotes the number of neural network layers. Table 9 illustrates the evaluation results (Top-1 and Top-5 accuracy) of the DenseNet model on ImageNet [55].

#### 3.4.6. Inception-ResNet-v2 Model

The Inception-ResNet-v2 model [57] is a convolutional neural architecture that builds from 164 layers and is trained on 1000 objects. The main purpose of the Inception-ResNet-v2 model is to achieve high results and better performance with low computational time. Table 10 illustrates the evaluation results (Top-1 and Top-5 accuracy) of the Inception-ResNet model on the ImageNet [55] dataset.

#### 3.4.7. Transfer Learning

**Figure 5:**
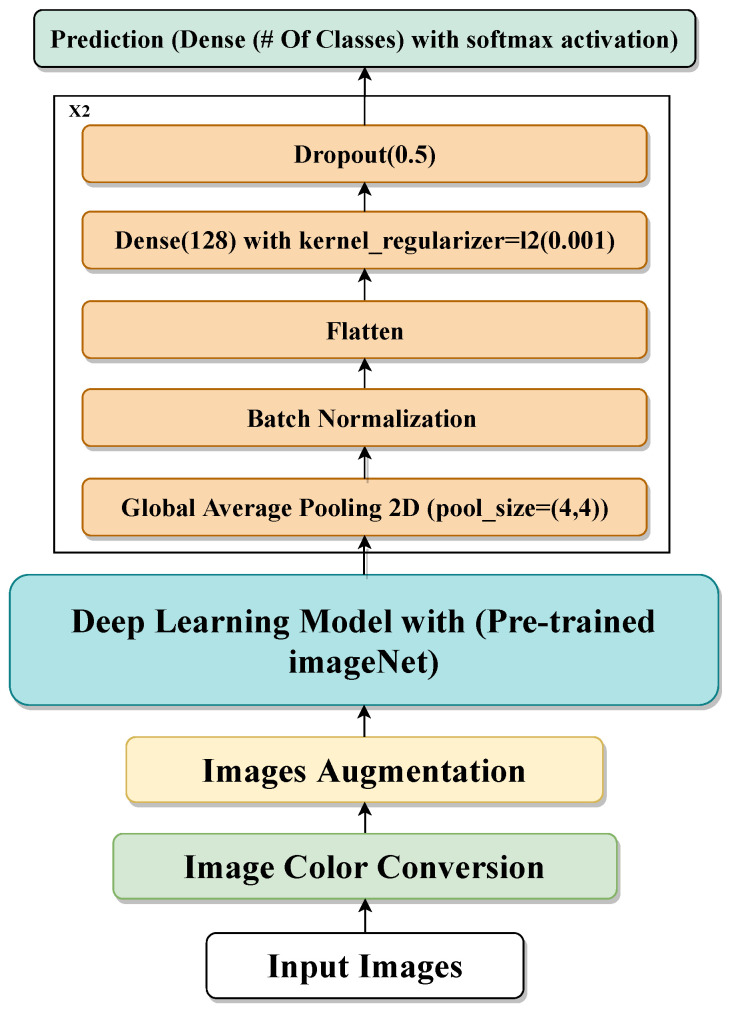
Proposed transfer learning-based technique.

Transfer learning [58] is a machine learning technique based on the re-use of existing pre-trained models that are trained on large-scale datasets for new tasks. Using the transfer learning technique for your dataset means you re-use the feature extraction part from the pre-trained model, which means our model just does the classification part and we do not need to extract the feature part. In our proposed model, we use transfer learning based on various pre-trained models, one at a time, like Xception, Inception-V3, ResNet50, VGG19, DenseNet201, and InceptionResNet-v2. Then, we feed the extracted feature into many layers/models: Average-Pooling2D, Flatten, Dense, and Dropout. We use Dense with the ‘softmax’ activation function as the output layer. Figure 5 shows the transfer learning of the proposed model.

## 4. Results

In this section, firstly we discuss the hyper-parameters used to fine-tune the proposed models for the pneumonia detection process in Section 4.1; secondly, we present the evaluation measures used to evaluate the proposed models in Section 4.2 for both the binary and the multi-class classification tasks. Finally, we discuss the models’ results for both tasks, in Section 4.3.

### 4.1. Experiments Setup and Model Parameters

Table 11 illustrates the hyper-parameters used to train the proposed models for the detection of pneumonia for both tasks. PR represents the number of parameters of the model, LR represents the learning rate, O represents the optimizer, EP represents the number of epochs used to train the models, BS represents the batch size, IS represents the size of the input images, and CH represents the number of channels of the input images.

### 4.2. Evaluation Measures

In this research, we utilized four different measures to evaluate the performance of the proposed models, regarding the pneumonia process.

#### 4.2.1. Accuracy

Accuracy is one of the well-known measures used to evaluate any machine or deep learning model by calculating the number of correctly and incorrectly classified instances. The accuracy measure can be computed using Equation (1):

(1) $$Accuracy\left(ACC\right)=\frac{TP+TN}{TP+TN+FP+FN}$$

#### 4.2.2. Precision

Precision is a measure that represents the number of instances that the model predicted correctly out of the overall predicted instances. The precision measure can be computed using Equation (2):

(2) $$Precision=\frac{TP}{TP+FP}$$

#### 4.2.3. Recall

Recall is a measure that represents the number of instances that the model predicted correctly out of the overall instances in the dataset. The recall measure can be computed using Equation (3):

(3) $$Recall=\frac{TP}{TP+FN}$$

#### 4.2.4. F1-Score

The F1-Score is a measure computed on the basis of precision and recall measures, using the following Equation (4):

(4) $$F1=2*\frac{\left(Precision*Recall\right)}{\left(Precision+Recall\right)}$$

where

True Positives (*TPs*) can be defined as cases in which the predicted diagnosis is Normal and the actual diagnosis is also Normal;

True Negatives (*TNs*) can be defined as cases in which the predicted diagnosis is Abnormal and the actual diagnosis is also Abnormal;

False Positives (*FPs*): can be defined as the cases in which the predicted diagnosis is Normal and the actual diagnosis is Abnormal; and

False Negatives (*FNs*) can be defined as the cases in which the predicted diagnosis is Abnormal and the actual diagnosis is Normal.

### 4.3. Results

Table 12 presents the results for binary classification, showing the five models utilized to classify the X-ray images as Normal or Abnormal using the data-augmentation technique. The results show that the proposed Xception model with data augmentation achieved the best performance, with 98.13%, 98.14%, 97.65%, and 97.89% for accuracy, precision, recall, and F1-score, respectively. From Table 12, we observe that the high precision of the Xception model (98.14%) suggests that the model excels at minimizing False Positives, which is crucial in medical imaging analysis, to avoid misdiagnosing healthy patients, while for DenseNet-201’s model the reported recall (94.70%) shows that it performed well in identifying Abnormal cases but that its performance scores were slightly lower than the Xception model’s, in terms of accuracy and the F1-score.

Table 13 illustrates the results for the six models used for the multi-class classification task and to classify the X-ray images as Normal, COVID-19, or Pneumonia, using a data-augmentation technique. The results show that the proposed Xception model with data augmentation outperformed the other models but showed reduced performance compared to binary classification, with 87.7%, 90.20%, 87.73%, and 87.49% for accuracy, precision, recall, and F1-score respectively. For the obtained results shown in Table 13, we observe that the Xception model achieving 90.20% precision highlights its ability to correctly identify COVID-19 cases, which is vital for medical imaging analysis and pandemic management, while Inception-ResNet-V2 demonstrated similar accuracy but lagged slightly behind in recall, suggesting a potential overfitting to specific class features.

## 5. Discussion

### 5.1. Model Performance and Comparison with Existing Studies

The results from this study demonstrate that the Xception model outperformed the other utilized deep learning models in both binary and multi-class classification tasks. With binary classification accuracy of 98.13%, the Xception model showed strong generalization abilities compared to the other models, like ResNet-50 and Inception-V3, which are traditionally effective in medical image analysis. These findings are consistent with the state-of-the-art research shown in Section 2. For the multi-class classification, the performance of Xception was slightly reduced to 87.7% accuracy, but the obtained results still highlight the ability of Xception to handle more complex tasks. Several studies have reported similar challenges with multi-class classification for different pneumonia types and other lung infections, often resulting in decreased model performance, as in [29]. Compared to recent studies, the results obtained in this research demonstrate competitive performance. These comparative results indicate that while several state-of-the-art techniques utilize complex multi-model architectures or novel optimization methods our proposed approach remains competitive, with a focus on simplicity, transfer learning, and effective data augmentation, suggesting that it is more generalizable and reliable in real-world scenarios.

### 5.2. The Role of Data Augmentation

The extensive utilization of data augmentation in this study contributed to the robustness of the proposed model. The results obtained from the multiple augmentation techniques expanded the dataset, ultimately allowing the models to be more generalized. Several previous works have also shown that data augmentation is critical when dealing with smaller datasets, especially in the medical domain, where in many cases having large-scale, diverse datasets can be difficult. Moreover, the usage of augmented images could mimic real-world variations in medical imaging, enabling the models to perform more robustly in real-world clinical conditions. This finding aligns with several existing studies that suggest augmentation can improve the diversity of training data and prevent overfitting [59,60].

### 5.3. Real-World Application and Clinical Integration

As shown by many prior studies about the integration of CNN models into the existing diagnostic workflows, as [61], the proposed model has the potential to be added to the clinical routine. Radiologists could utilize the developed model to obtain a diagnostic suggestion with high accuracy, as the model can perform well in binary and multi-class classifications with high precision (98.14% for binary classification and 90.20% for multi-class). This makes them ideal candidates for deployment as helping tools in emergency departments and radiology units. This deployment could streamline decision making in clinical settings, reduce the workload of radiologists, and improve patient outcomes through faster and more accurate diagnosis. Despite the significant performance of the proposed model, there are still several challenges and limitations that must be addressed, for successful clinical deployment. Regulatory approval from healthcare authorities would be essential to ensuring compliance with safety standards. Additionally, transparency and explainability are critical, especially in medical AI applications. Integrating explainable AI (XAI) techniques, such as Grad-CAM [62], could provide visual explanations of model works and decisions, which could enhance clinician acceptance and trust and facilitate ML and DL techniques adoption in clinical environments.

### 5.4. Limitations and Future Directions

An important factor that was explored in this study, and which needs to be highlighted, is the timing of image acquisition relative to disease progression. Research, such as [63], suggests that COVID-19 demonstrates differently at the different disease progression stages, which may affect the model’s ability to accurately classify images. For instance, early-stage COVID-19 may present with fewer or more subtle radiological signs than advanced-stage infection, potentially leading to misclassification. Future work could be to incorporate metadata, such as the time since symptom onset, into the ground truth process. This additional contextual information could help models differentiate between early-stage and advanced cases, which may improve classification accuracy. Another factor to explore is the model’s interpretability, which remains a key challenge, particularly for medical applications. In future work, robust interpretability techniques, such as attention mechanisms or Grad-CAM, could be employed to enhance clinician trust. This could allow healthcare professionals to understand how and why a model makes a specific diagnosis, increasing transparency and clinical applicability.

## 6. Conclusions

In this research, we proposed several deep learning models for solving two classification tasks, using X-ray images collected from two public resources. The first task was the binary classification task, which classified X-ray images as Normal or Abnormal. The second task was a multi-class classification task, which classified X-ray images into pneumonia, normal, or COVID-19. We applied several data-augmentation techniques. By evaluating the models in this research, the Xception model outperformed the other proposed models in the two tasks. The binary task achieved accuracy, precision, recall, and an F1-score of 98.13%, 98.14%, 97.65%, and 97.89%, respectively, whereas the results for the multi-classification tasks were 87.73%, 90.20%, 87.73%, and 87.49%, respectively. For future work, we plan to increase the size of the dataset by using other resources. We also plan to use other deep learning models, like efficient-net, and to compare their performance with the used models.

## Figures and Tables

**Table 2:** Binary classification task dataset information.

Class	# Train Images	# Test Images
Normal	4500	500
Abnormal	9000	1000

**Table 3:** Multi-class classification task dataset information.

Class	# Train Images	# Test Images
Normal	4500	500
COVID-19	4500	500
Pneumonia	4500	500

**Table 4:** Augmentation techniques description.

Augmentation Technique	Description
Scaling	We resized the images to 80–120% of their size.
Rotation	We rotated the image from −45 to +45 degrees.
Shearing	We transformed the orientation of the image.
Elastic and Perspective Transformation	We moved pixels around locally with random strengths.
Ordering	We used the nearest neighbor or bilinear interpolation.
Gaussian Blur	We blurred images, using a sigma between 0 and 3.0.
Average Blur	We blurred images, using local means with kernel sizes between 2 and 7.
Median Blur	We blurred images, using local medians with kernel sizes between 2 and 7.
Sharpen	We changed the lighting of the image by changing the color distribution.
Emboss	We changed the strength value.
Additive Gaussian Noise	We added Gaussian noise to images.
Multiplication	We changed the brightness of images, from −10 to 10 of the original value.
Addition	We changed the brightness of images, from 50 to 150% of the original value.
Horizontally Flipped	We horizontally flipped 50% of all the images.
Vertically Flipped	We vertically flipped 20% of all the images.

**Table 5:** Achievements of Xception model with parameters.

Version	Top-1 Acc.	Top-5 Acc.	# Parameters	Depth
Xception	79.0%	94.5%	22.9 M	126

**Table 6:** Achievements of Inception-V3 model with parameters.

Version	Top-1 Acc.	Top-5 Acc.	# Parameters	Depth
inception-V3	77.9%	93.7%	23.8 M	159

**Table 7:** Achievements of ResNet model with parameters.

Version	Top-1 Acc.	Top-5 Acc.	# Parameters	Depth
ResNet-50	74.9%	92.1%	25.6 M	-
ResNet-101	76.4%	92.8%	44.7 M	-
ResNet-152	76.6%	93.1%	60.4 M	-

**Table 8:** Achievements of VGG model with parameters.

Version	Top-1 Acc.	Top-5 Acc.	# Parameters	Depth
VGG16	71.3%	90.1%	138.3 M	23
VGG19	71.3%	90.0%	143.6 M	26

**Table 9:** Achievements of DenseNet model with parameters.

Version	Top-1 Acc.	Top-5 Acc.	# Parameters	Depth
DenseNet-169	76.2%	93.2%	14.3 M	169
DenseNet-121	75.0%	92.3%	8.1 M	121
DenseNet-201	77.3%	93.6%	20.2 M	201

**Table 10:** Achievements of Inception-ResNet-v2 model with parameters.

Version	Top-1 Acc.	Top-5 Acc.	# Parameters	Depth
Inception-ResNet	80.3%	95.3%	55.8 M	572

**Table 11:** Model hyper-parameters for both binary and multi-class classification tasks.

Model	PR	EP	BS	LR	O	IS	CH
DenseNet	20.2 M	100	16	1 × 10^−4^	Adam	224 × 224	3
VGG	138.3 M	100	16	1 × 10^−4^	Adam	224 × 224	3
ResNet-50	25.6 M	100	16	1 × 10^−4^	Adam	224 × 224	3
Inception-V3	23.8 M	100	16	1 × 10^−4^	Adam	299 × 299	3
Inception-ResNet-V2	55.8M	100	16	1 × 10^−4^	Adam	299 × 299	3
Xception	22.9 M	100	16	1 × 10^−4^	Adam	299 × 299	3

**Table 12:** Binary classification task results.

Models	Accuracy	Precision	Recall	F1-Score
DenseNet-201	95.46%	95.07%	94.70%	94.88%
VGG-16	88.53%	89.60%	84.40%	86.26%
ResNet-50	96.93%	96.55%	96.55%	96.55%
Inception-V3	97.00%	96.60%	96.65%	96.63%
Inception-ResNet-V2	93.67%	94.52%	91.25%	92.62%
**Xception**	**98.13%**	**98.14%**	**97.65%**	**97.89%**

**Table 13:** Multi-classification result.

Models	Accuracy	Precision	Recall	F1-Score
DenseNet-201	87.5%	90.00%	88.00%	87.00%
VGG-16	74.80%	76.0%	75.00%	75.00%
ResNet-50	78.40%	90.00%	87.00%	87.00%
Inception-V3	86.50%	89.00%	78.00%	86.00%
Inception-ResNet-V2	87.1%	89.00%	87.00%	87.00%
**Xception**	**87.7%**	**90.20%**	**87.73%**	**87.49%**

## Data Availability

Data used in this research are deposited in a repository and are publicly available.
